# Doxycycline and therapeutic targeting of the DNA damage response in cancer cells: old drug, new purpose

**DOI:** 10.18632/oncoscience.215

**Published:** 2015-08-24

**Authors:** Maria Peiris-Pagès, Federica Sotgia, Michael P. Lisanti

**Affiliations:** ^1^ The Breast Cancer Now Research Unit, Institute of Cancer Sciences, University of Manchester, Manchester, UK; ^2^ The Manchester Centre for Cellular Metabolism (MCCM), Institute of Cancer Sciences, University of Manchester, Manchester, UK

**Keywords:** doxycycline, drug repurposing, DNA-PK, DNA damage response, antibiotics for cancer therapy, cancer stem cells, mitochondria, cancer metabolism

## Abstract

There is a small proportion of cells within a tumour with self-renewing properties, which is resistant to conventional therapy, and is responsible for tumour initiation, maintenance and metastasis. These cells are known as cancer stem cells (CSCs) or tumour-initiating cells (TICs) [1]. Recent publications identify several antibiotics, such as salinomycin or doxycycline, as selective CSCs inhibitors [2-4]. However, the mechanisms of action of these antibiotics on CSCs are not fully understood.

## Doxycycline effects on DNA repair

**Figure 1:**
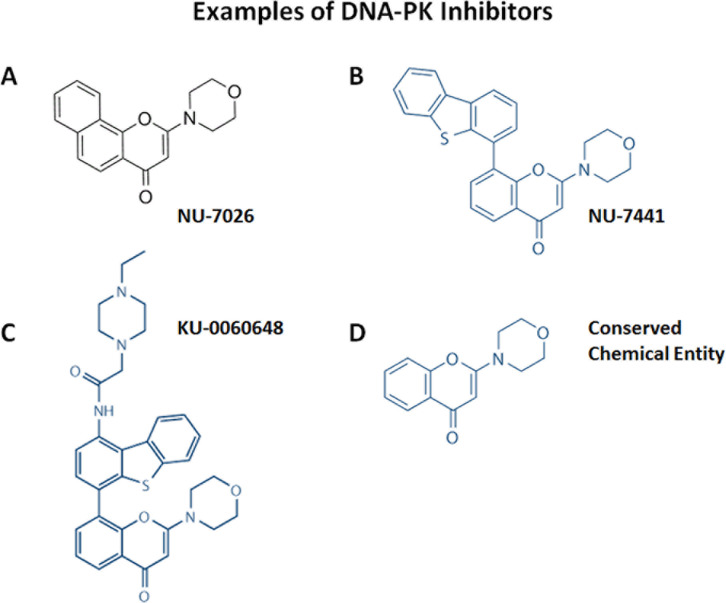
Chemical structures of three known DNA-PK inhibitors A) NU-7026, B) NU-7441, C) KU-0060648 and D) a chemical entity common to all of these DNA-PK inhibitors

**Figure 2:**
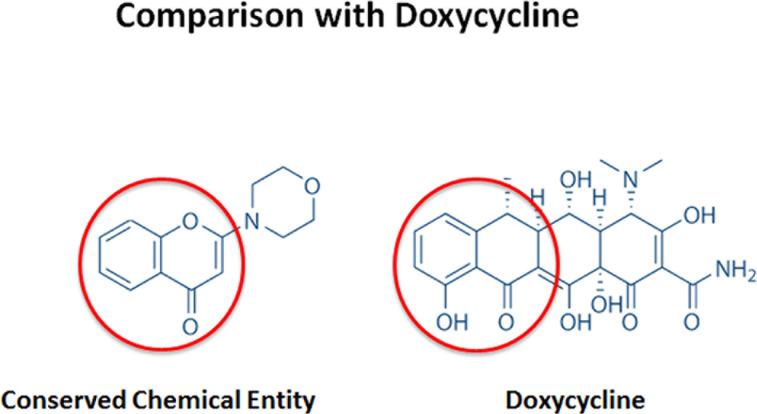
Chemical structure and comparison between doxycycline and a conserved chemical entity within DNA-PK inhibitors Doxycycline is a reduced carba-analogue of other DNA-PK inhibitors.

Previous studies performed in our lab showed higher levels of mitochondrial proteins in breast CSCs, defined by their capability to grow in suspension as mammospheres, compared with the bulk of cancer cells grown under regular conditions [2]. That discovery led to the hypothesis that such increase in mitochondrial protein abundance could be reversed by treating these mammospheres with antibiotics, hence eradicating the CSCs population, as several classes of FDA-approved antibiotics can inhibit mitochondrial protein synthesis. Indeed several antibiotics, such as doxycycline, were able to inhibit mammosphere formation [2]. However, the mechanism of action of doxycycline remained unidentified and in a further effort to elucidate it, our lab recently published a report demonstrating reduced expression of proteins associated with mitochondrial metabolism, EMT, protein synthesis, and more curiously, with DNA damage response after doxycycline treatment in a breast cancer cell line [5]. Particularly, one of the best doxycycline targets identified by our quantitative proteomics analysis was DNA-PK, the catalytic subunit of the DNA-dependent protein kinase, which is required for proper NHEJ (non-homologous end-joining) DNA repair [6], for maintenance of mitochondrial DNA integrity and copy number [7], and it confers resistance to radiation and chemotherapy in cancer cells [8]. Interestingly, DNA-PK was also found to be up-regulated in mammospheres, and its genetic knock-down or pharmacological inhibition using either doxycycline or an established DNA-PK inhibitor (NU7441) blocked that mammosphere formation. In fact, a closer look at the chemical structure reveals that doxycycline is a reduced carba-analogue of other DNA-PK inhibitors (Figures 1 and 2), although the direct interaction between doxycycline and DNA-PK still needs to be proved. Mechanistically, doxycycline treatment reduced not only the general metabolic state of breast cancer cells and their capacity to resist anoikis, but also inhibited their antioxidant response and several stem-related signalling pathways including Wnt, Shh, Notch, TGFβ and STAT3, the inhibition of which induces anoikis and radio and chemosensitivity [1, 9-14]. DNA-PK has even been shown to directly interact with LEF1, which acts downstream in the Wnt signaling [15]. Similarly, salinomycin, another antibiotic, has also been reported to act on breast CSC by inhibiting Wnt pathway [3].

Radiation and chemotherapy induce DNA damage, which is lethal to the cells if it is not repaired. It is now known that CSCs protect themselves from DNA damaging treatment by regulating cell cycle, by scavenging more efficiently the reactive oxygen species and by enhancing their DNA repair capability. Several studies demonstrate that CSCs have an activated DNA repair process, as well as a more robust activation of DNA damage response genes and proteins than non-stem cells [16], which makes them resistant to cancer therapy. In line with these results, our study also shows the effects of doxycycline on increasing sensitivity to radiation treatment.

## Doxycycline effects on neddylation and ubiquitination

Recent clinical trials involving doxycycline have already shown positive therapeutic effects in lymphoma patients [17]. In agreement with that, a recent publication by Pulvino et al., demonstrated that doxycycline inhibits diffuse large B cell lymphoma growth *in vitro* and *in vivo*, in part via inhibition of CSN5 activity and reduction of HSP90 levels and function [18]. CSN5 is a member of the COP-9 signalosome complex that inhibits neddylation, a process similar to ubiquitination. Genetic or pharmacological inhibition of CSN5 using doxycycline was able to decrease CSN5 deneddylation function, impairing cell survival in diffuse large B cell lymphoma cells. Altogether these results indicated that doxycycline treatment induces proteasomal degradation by increasing ubiquitination and neddylation in lymphoma cells. In addition, the study demonstrated that doxycycline treatment causes cell cycle arrest and inhibits NFkB and STAT3 signalling, as our experiments show.

Finally, in the report the authors mention that they observed synergetic cytotoxic effects of doxycycline and several chemotherapeutic agents in diffuse large B cell lymphoma, giving another indication that doxycycline targets chemoresistant CSCs. In contrast with that, though, it has been shown in human cells that inhibition of neddylation sensitizes cells to radiotherapy and chemotherapeutic agents although the mechanisms behind this synergy are not fully understood and have not been consistent [19]. Nevertheless, it is now well established that an enhanced DNA damage response is one of the features that allow CSCs to overcome treatment. The efficiency of DNA damage detection and repair requires the recruitment and modification of a complex protein network. The DNA damage response is a tightly regulated process involving reversible post-translational modifications such as ubiquitination and neddylation, which regulate protein stability and function, preventing *de novo* protein synthesis [16, 19].

To sum up, in addition to the well-known doxycycline anti-microbial and anti-inflammatory effects, and its ability to inhibit metal membrane proteinases (MMPs) by chelating the essential zinc ions of these enzymes [20], this new evidence in breast cancer and lymphoma cells indicate that doxycycline has also a clear impact in regulating the DNA damage response, including DNA repair (DNA-PK), ubiquitination and neddylation (CSN5 and HSP90), as well as in suppressing different developmental and EMT pathways (Wnt, Notch, Hedgehog, TGFβ and STAT3), all processes found to be reinforced in CSCs and to confer resistance to anoikis and conventional therapies [1, 9, 11-14]. A successful cancer therapy should aim to eliminate all cancer cells, including the intrinsically resistant CSC population, which ultimately will give rise to recurrence and metastasis. That is why the concept that doxycycline and other antibiotics may reduce the viability and growth of the CSC population is of great importance and should be further investigated.

Last but not least, doxycycline is an excellent example of how existing, inexpensive, well-tolerated drugs might be repurposed not only as new cancer therapeutic agents, but also might provide the new insights needed to fully understand CSC traits in tumours. Likewise it is important to properly establish the bioequivalent dose required to block CSCs.
